# Cerebellar Coordination of Neuronal Communication in Cerebral Cortex

**DOI:** 10.3389/fnsys.2021.781527

**Published:** 2022-01-11

**Authors:** Samuel S. McAfee, Yu Liu, Roy V. Sillitoe, Detlef H. Heck

**Affiliations:** ^1^Department of Diagnostic Imaging, St. Jude Children’s Research Hospital, Memphis, TN, United States; ^2^Department of Anatomy and Neurobiology, University of Tennessee Health Science Center, Memphis, TN, United States; ^3^Department of Pathology and Immunology, Baylor College of Medicine, Houston, TX, United States; ^4^Department of Neuroscience, Baylor College of Medicine, Houston, TX, United States; ^5^Development, Disease Models & Therapeutics Graduate Program, Baylor College of Medicine, Houston, TX, United States; ^6^Jan and Dan Duncan Neurological Research Institute of Texas Children’s Hospital, Houston, TX, United States

**Keywords:** cerebellum, cerebrocerebellar communication, coherence, functional connectivity, cognition

## Abstract

Cognitive processes involve precisely coordinated neuronal communications between multiple cerebral cortical structures in a task specific manner. Rich new evidence now implicates the cerebellum in cognitive functions. There is general agreement that cerebellar cognitive function involves interactions between the cerebellum and cerebral cortical association areas. Traditional views assume reciprocal interactions between one cerebellar and one cerebral cortical site, *via* closed-loop connections. We offer evidence supporting a new perspective that assigns the cerebellum the role of a coordinator of communication. We propose that the cerebellum participates in cognitive function by modulating the coherence of neuronal oscillations to optimize communications between multiple cortical structures in a task specific manner.

## Introduction

Higher order brain functions, including cognitive processes, involve precisely coordinated neuronal communications between multiple cerebral cortical structures (e.g., Damasio, 1989; Vaadia et al., 1995; Mesulam, 1998; Ayzenshtat et al., 2010). In a seminal publication, Fries (2005) proposed a mechanism for controlling neuronal communications between brain structures through the modulation of coherence of their neuronal oscillations (Box 1). Experimental findings have since provided substantial support for the concept of “communication through coherence” (CTC), showing that coherence changes do indeed correlate with changes in the effectiveness of neuronal transmission, and that coherence changes occur in a task-specific manner. CTC has been studied in detail in the context of decision making. In rodents, decision-making in SWM requires the coordinated activity of the medial prefrontal cortex (mPFC) and dorsal hippocampus (Churchwell and Kesner, 2011; Gordon, 2011). Simultaneous electrophysiological recordings in the mPFC and hippocampus during performance of SWM tasks have shown that the decision process is associated with an increase in the coherence of theta oscillations between the mPFC and dorsal hippocampus (Jones and Wilson, 2005; Hyman et al., 2010; Benchenane et al., 2011; Gordon, 2011). A comparison of correct and incorrect decisions revealed that mPFC-hippocampal theta coherence reached higher values during correct compared to incorrect decisions, supporting a functional role of coherence in this task (Jones and Wilson, 2005; Hyman et al., 2010). In order to affect brain function changes in coherence need to affect changes in spike activity. In the context of SWM two studies measured both spike activity and local field potential (LFP) coherence and showed that an increase in coherence is accompanied by an increase in entrainment of mPFC spike activity to the phase of the coherent mPFC-hippocampal theta oscillations (Jones and Wilson, 2005; Hyman et al., 2010). For additional examples of experimental support for CTC, including an influence of coherence on spike activity see also (Jones and Wilson, 2005; Siegel et al., 2008; Bosman et al., 2012; Brunet et al., 2014; Sigurdsson and Duvarci, 2016; Bonnefond et al., 2017; Palmigiano et al., 2017; McAfee et al., 2018).

**Box 1. Fundamental principles of the Communication Through Coherence (CTC) theory, and their extension to account for cerebrocerebellar interactions.**

– Gamma oscillations (>30 Hz) are generated through rhythmic sequences of excitation and inhibition within a local group of neocortical neurons, creating brief temporal windows of high and low excitability.
– Communication between neuronal groups is most effective when the output of the presynaptic group is aligned with a high-excitability window of the postsynaptic group. Synchronization in the gamma range facilitates this.
– A neuronal group receiving gamma-rhythmic inputs from several different presynaptic groups will preferentially respond to the group best aligned with its high-excitability windows, thereby providing selective communication.
– Selective synchronization of gamma is influenced by “top-down” signals that are typically in the alpha/beta range (5–30 Hz). Alpha is typically inhibitory, but beta can enhance gamma frequency to aid in selective synchronization.
– Gamma amplitude is highest in the supragranular layers which tend to direct their influence to higher cortices. Alpha/beta amplitude is highest in the infragranular layers, which project to lower cortices as well as the cerebellum via the pontine nuclei.
– We propose that the cerebellum encodes rhythms in the alpha/beta range that reflect the topographical pattern of gamma activation in the cerebral cortex and generates feedback to facilitate appropriate gamma-rhythmic synchronization in communicating neuronal groups.
– This gamma-rhythmic synchronization may be accomplished via the direct induction and modulation of neocortical gamma, or the indirect modulation of gamma through alpha/beta rhythms.

Importantly, the CTC theory describes a mechanism for *flexibility* in communication between neuronal groups that allows for selective information flow but does not explain the neuronal mechanism for this selectivity itself. The CTC theory proposes that “top-down” signals arise to modulate the effective transmission from “bottom-up” sources of sensory information, with “top-down” signals emerging as a consequence of internally maintained processes such as cognition or attention. The source(s) of these signals remains unknown in many cases. What is perhaps the most intriguing uncertainty is how changes in coherence selectively occur to result in the appropriate spatiotemporal synchronization for a given task. We propose that this process requires the cerebellum as a coordinator of task specific communication, a role that is consistent with existing interpretations of cerebellar function, like supervised learning and internal modeling of sensory and motor functions.

There is extensive evidence for cerebellar involvement in cognitive functions, such as language processing, working memory, and executive function (Marvel and Desmond, 2010; Brissenden et al., 2018; Ashida et al., 2019; Heleven et al., 2019). Anatomical and imaging studies show extensive connections between the cerebellum and neocortical areas essential for cognitive functions in healthy brains (Ito, 2008; Strick et al., 2009; Buckner, 2013). Posterior fossa syndrome, a condition that often develops after surgical removal of a medulloblastoma – a brain tumor that develops in the posterior fossa region of the brain – is characterized by impairments of cognition, emotion, and expressive language (Schmahmann et al., 2007; Morris et al., 2009). Patay (2015) suggested that the severity of posterior fossa syndrome is determined by the degree of damage to the cerebrocerebellar connection pathways during surgery, rather than to the extent of cerebellar damage (Patay, 2015). The sheer spectrum of cognitive functions now linked to the cerebellum (Rapoport et al., 2000; Schmahmann et al., 2019) suggest that the cerebellar contribution supports a general neuronal principle of cognitive processes rather than a specific contribution to any individual particular function.

Thus, accepting a central, albeit yet undefined role of the cerebellum in cognition, progress toward a complete understanding of normal cognitive brain function and of the neuropathology of mental illnesses must include a more comprehensive understanding of the neuronal mechanisms that comprise the cerebellar involvement in cognition.

Even before there was broad acceptance of a cerebellar role in cognition, it became obvious that cerebellar neuropathology was one of the most common neuropathologies found in the brains of patients with autism spectrum disorder (ASD) (Bauman and Kemper, 1985; Courchesne, 1997; Fatemi et al., 2012; Becker and Stoodley, 2013) or schizophrenia (Weinberger et al., 1980; Jurjus et al., 1994; Picard et al., 2007; Andreasen and Pierson, 2008). More recently, studies have also implicated the cerebellum in dementia and Alzheimer’s disease (Schmahmann, 2016; Jacobs et al., 2018). As we review below, these diseases are often associated with changes in coherence of cortical oscillations, indicative of dyscoordination of communication consistent with the cerebellum failing to perform its proposed role as a coordinator of communication.

**FIGURE 1:**
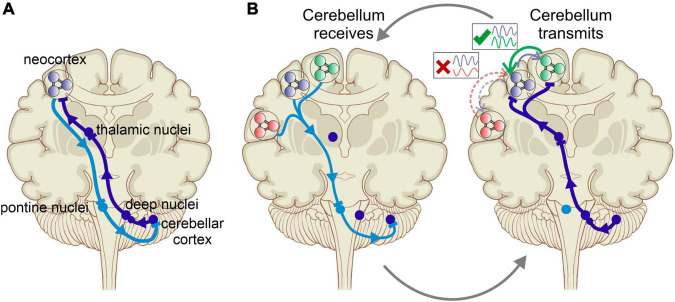
**(A)** Cerebrocerebellar interaction *via* reciprocal connections between specific cerebral and cerebellar areas. Purkinje cells in the cerebellar cortex project to the neocortical areas *via* the cerebellar nuclei and the thalamus. In what was often described as a closed-loop projection, the neocortex in turn projects back to the cerebellar area of origin *via* the pontine nuclei. This one-to-one interaction scheme is the basis of most approaches to investigating cerebrocerebellar interactions. **(B)** Cerebellar modulation of communication between cerebral cortical areas provides a revised picture of cerebrocerebellar interactions, in which the cerebellum does not primarily modulate the activity in specific cortical areas but rather coordinates the communication between areas by augmenting the coherence of neuronal oscillations in a task specific manner. This occurs cyclically with the cerebellum areas receiving the neuronal “context” of cerebral activity from multiple regions by encoding their oscillations, comparing their timing, and then transmitting the output *via* the thalamus to promote synchrony between task-appropriate cerebral cortical regions.

The new perspective we propose here reconciles some of the prevailing theories in cerebral and cerebellar research. Tracing cerebrocerebellar connectivity using transneuronal transport of neurotropic viruses revealed reciprocal connections between a specific cerebellar region and a specific cerebral cortical site, suggesting a separation of function through closed-loop connections (Middleton and Strick, 2001; Kelly and Strick, 2003; Figure 1A). However, newer studies documented considerable convergence and divergence in cerebrocerebellar connectivity, painting a more complex picture that allows for a richer interaction between structures and functions (Henschke and Pakan, 2020). The latter view is more in line with the proposed new perspective of the cerebellum as a coordinator of task-specific neuronal communication between cerebral cortical structures *via* the modulation of coherence of oscillations (Figure 1B). We propose, based on recent experimental findings from our labs (McAfee et al., 2019) and from others (Popa et al., 2013; Lindeman et al., 2021), that the cerebellum accomplishes this by encoding the phase relations of ongoing neuronal oscillations in neocortical areas and providing task-appropriate feedback that promotes specific spatiotemporal patterns of gamma activation. Ultimately, these interactions provide “top-down” selectivity for inter-areal coherence.

Modulation of coherence is a temporal coordination task, requiring similar millisecond-range precision as the temporal coordination of muscle contractions for motor control, for which the cerebellum is known to be crucially important. The unique cerebellar cortical network architecture and cellular properties ideally enable the cerebellum to encode neocortical oscillations and transform this information into task-specific outputs to modulate coherence. This new perspective of cerebrocerebellar interaction also sheds a new light on findings from imaging studies that have identified cerebellar loci as parts of brain-wide networks (Habas et al., 2009; Buckner et al., 2013; Halko et al., 2014; Guell et al., 2018a,b). Assuming a role of the cerebellum as coordinator of cerebral cortical communication, a new approach is to link activity in the cerebellar nodes to the strength of functional connectivity between cerebral cortical nodes of the network. Recent experiments by Halko et al. (2014) provide some support for this notion, showing that stimulation of the cerebellar cortex in humans increased functional connectivity in the default mode network. Looking at known functional and anatomical cerebrocerebellar connectivity patterns with this new perspective provides new opportunities for resolving key questions around the neuronal “language” of cerebrocerebellar interactions.

## Dynamic Coordination of Neuronal Activity in the Cerebral Cortex

Cerebral functional networks are defined as such based on robust and consistent spatiotemporal patterns of neuronal activity, often linked to specific brain states and mental operations (Fox and Raichle, 2007; Ayzenshtat et al., 2010; Raichle, 2015). These patterns are manifest in different ways at varying spatial and temporal scales, resulting in distinct but interrelated observations with different imaging modalities. Brain-wide functional networks identified with functional MRI reflect spatial patterns of neuronal activity that is temporally coordinated on the scale of hundreds of milliseconds to seconds. The vasodilation that drives these BOLD signal patterns in the neocortex is tightly linked to the bursting of gamma oscillations (Mateo et al., 2017), which are highly focal in nature and influence communication on the neuronal level (Fries, 2005). Oscillations in the alpha and beta range (5–30 Hz) are more spatially diffuse and effect both the occurrence and coherence of gamma oscillations (Richter et al., 2017). Resting state brain networks can also be captured using magnetoencephalography (MEG), which provides a higher temporal resolution and allows capturing oscillations at higher frequency bands, including alpha and beta rhythms. Brookes et al. (2011) used MEG measurements to recreate the spatial patterns that constitute functional networks in fMRI. This involvement of alpha and beta oscillations in brain wide functional networks together with their modulation of gamma oscillations suggests that they may play a key role in the spatial selectivity of gamma coherence, forming a critical link between communication at the neuronal level and the macroscopic organization of brain-wide functional networks.

In the following sections, we will review evidence that the cerebellum is essential for the coherence of cerebral gamma oscillations within a well-defined functional network, and that the cerebellar activity reflects information about cerebral oscillations within a broad range of frequencies. We propose that these findings, along with a trove of anatomical, physiological, and imaging evidence, supports the idea that the cerebellum plays a key role in the modulation of gamma coherence across different areas of the cerebral cortex. We propose that this is accomplished through the encoding of sub-gamma cerebral oscillations by the cerebellum, and the subsequent generation of cerebello-cortical feedback. The result of this feedback is the modulation of cerebral gamma and thus its coherence, although it remains to be explored whether gamma is modulated directly or indirectly through the modulation of sub-gamma oscillations.

### Experimental Evidence Supporting Cerebellar Coordination of Communication by Coherence

**FIGURE 2:**
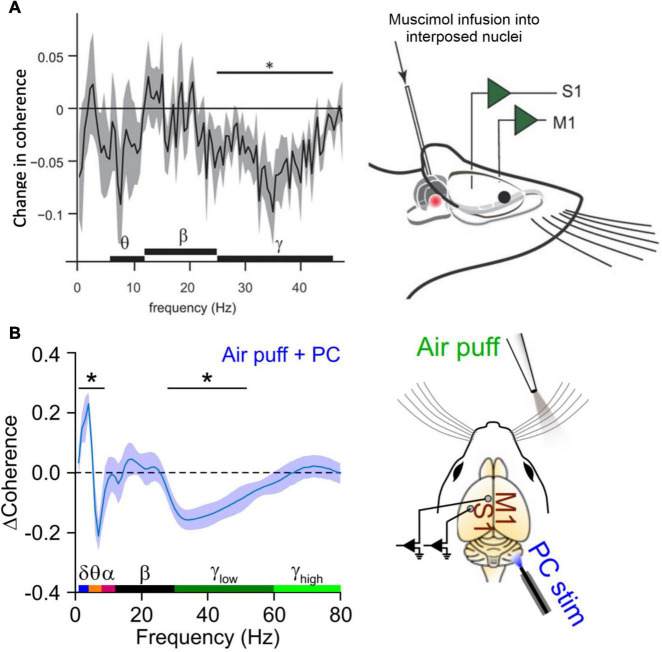
Inactivation of cerebellar output reduces gamma coherence between S1 and M1. **(A)** In an experiment that involved simultaneous measurements of LFPs in S1 and M1 of awake, head fixed rats, Popa et al. (2013) demonstrated that pharmacological inactivation of the interposed nuclei selectively reduced gamma coherence between S1 and M1. The plot on the left shows a change in coherence relative to the control condition in which the interposed was kept intact. The experimental approach is illustrated on the right (from: Popa et al., 2013). **(B)** A similar, recent experiment showing that optogenetic inhibition of cerebellar output (*via* Purkinje cell excitation) significantly reduced the coherence of gamma responses evoked by whisker stimulation. The plot on the left shows estimated effect of Purkinje cell stimulation on coherence between deep layer S1 and superficial layer M1. Theta-range S1–M1 coherence was enhanced with Purkinje cell stimulation (from: Lindeman et al., 2021). *These frequencies were statistically significant (*p* < 0.05).

A seminal study by Popa et al. (2013) was the first to suggest a role for the cerebellum in coordinating coherence in the sensorimotor system. They performed simultaneous recordings of neuronal oscillations in the primary sensory (S1) and primary motor cortices (M1) of the mystacial whisker system in freely moving rats. Up to eight electrodes were placed in each area to allow analysis of coherence within S1 and M1 as well as between the two areas. Whenever the rats engaged in active whisker movements, coherence of gamma oscillations within S1 and M1 increased for the duration of the behavior (Popa et al., 2013; Figure 2A). A crucial involvement of the cerebellum in this behavior-related coherence increase became clear when the authors used Muscimol to pharmacologically inactivate the interposed nucleus of the cerebellum, i.e., the nucleus that projects to the whisker system *via* the motor thalamus. Inactivation of the interposed nucleus eliminated the increase of S1-M1 gamma coherence during whisking behavior (Popa et al., 2013). Importantly, the generation of gamma oscillations within each structure was not altered by inactivating cerebellar output. Thus, the generation of gamma rhythms *per se* did not require an intact cerebellar output, but between-structure coherence of gamma did. A very recent study supported these findings using optogenetic excitation of Purkinje cells to silence cerebellar output and examined the resulting changes in coherence in greater anatomical and temporal detail. Lindeman et al. (2021) used linear silicon probes to record along the cortical depth of S1 and M1 during sensory stimulation delivered *via* an air puff to the whiskers. Concurrent optical stimulation of Purkinje cells in the contralateral cerebellar hemisphere caused temporary dampening of cerebellar output, resulting in the loss of sensory-evoked S1-M1 coherence in the gamma range (Figure 2B). The authors also showed that Purkinje cell stimulation reduced the amplitude of evoked local field potential (LFP) response to whisker stimulation predominantly in the deep cortical layers of both S1 and M1. This effect, as well as the disruption of gamma-band coherence, was largely rescued by delaying the onset of Purkinje cell stimulation by 20 ms relative to the air puff, indicating that the coherence modulation was mediated by a fast, ascending cerebellar pathway. Additionally, Purkinje cell stimulation was accompanied by an increase in S1-M1 coherence within the theta range regardless of concurrent whisker stimulation. This suggests that theta-band coherence was a direct result of the transient cerebellar stimulation and may reflect a mechanism of cerebellar-controlled sub-gamma neuronal activity capable of mediating gamma-band activity. The authors completed the study by creating an *in silico* laminar model of cerebellar, cortical, and subcortical interactions showing that coherent gamma activity likely flowed from S1 to M1, while coherent theta was a top-down signal flowing from M1 to S1 (Lindeman et al., 2021). This is intriguing given the proposed role of theta within the CTC hypothesis – that it acts as a gating rhythm in the target region that modulates the effectiveness of gamma-frequency transmission from a given source (Fries, 2015). The cerebellar stimulation in this study appeared to induce a consistent theta phase relationship with M1 leading S1, which we would not expect to promote gamma-band propagation from S1 to M1.

### Signals Received by the Cerebellum: Cerebellar Encoding of Cerebral Oscillations

The findings by Popa et al. (2013) and Lindeman et al. (2021) discussed above are consistent with our proposed role of the cerebellum as a coordinator of coherence, but they do not provide information about the neuronal activity in the cerebellum itself. In order to modulate cortical coherence effectively for a given task, it is essential that the cerebellum can encode the neuronal “context” elicited by the task. This likely includes an array of neuronal oscillations that are commonly observed throughout different sensorimotor (e.g., Baker et al., 1999; Watanabe and Kohn, 2015) and cognition-related cortices (e.g., Osipova et al., 2006; Myers et al., 2014), and which may be offset with meaningful delays. With the majority of subcortically projecting layer V pyramidal neurons sending collaterals to the pontine nuclei (Leergaard and Bjaalie, 2007; Suzuki et al., 2012), information about oscillatory activity throughout the cerebral cortex is likely to reach the cerebellum *via* its mossy fiber (MF) inputs.

**FIGURE 3:**
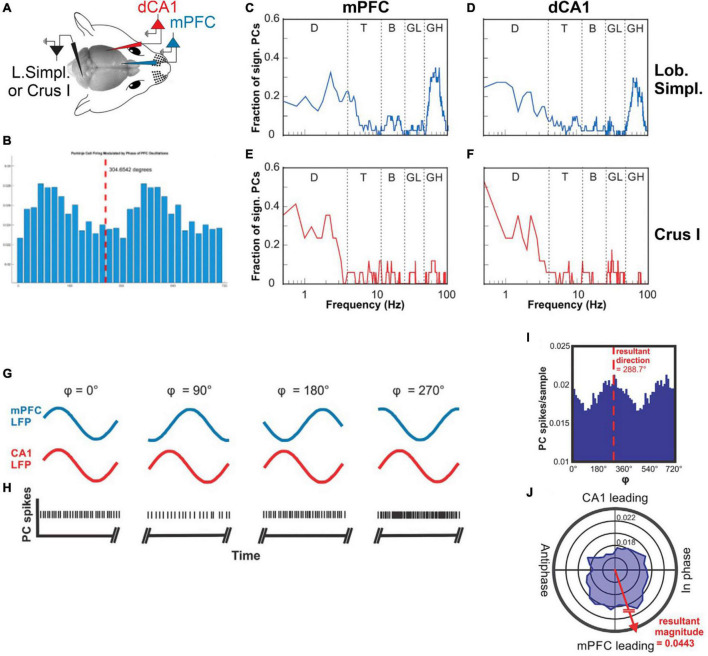
Cerebellar representations of phase and phase differences of oscillations in the mPFC and CA1. **(A)** Illustration of the experimental setup with recording electrodes in the mPFC and dCA1, picking up LFPs and a recording electrode in cerebellar lobulus simplex recording single unit Purkinje cell spike activity. **(B)** Example histogram showing Purkinje cell simple spike rate plotted against the phase of a 10 Hz oscillation recorded in the mPFC. **(C)** Fraction of Purkinje cells in LS (*n* = 32) whose simple spike activity was significantly correlated with oscillatory phase plotted as a function of mPFC oscillation frequency (plotted on a log-10 scale). The function shows two peaks at the delta frequency range (0.5–4 Hz) and the high gamma range (50–100 Hz). **(D)** As in **(C)** but showing fractions of LS Purkinje cells significantly modulated by the phase of oscillatory activity in CA1. **(E)** Fraction of Purkinje cells in Crus I (*n* = 17) whose simple spike activity was significantly correlated with the oscillatory phase in mPFC plotted as a function of mPFC oscillation frequency. The function shows a single peak at the delta frequency range (0.5–4 Hz). **(F)** As in **(E)** but showing fractions of Crus I Purkinje cells significantly modulated by the phase of oscillatory activity in CA1. D, delta; T, theta; B, beta; LG, low gamma; HG, high gamma. **(G)** Illustration of hypothetical oscillations at a specific frequency occurring simultaneously in the mPFC (blue traces) and CA1 (red traces) and displaying different phase relationships (4) at different times. The phase relationship 4 is defined as the phase difference relative to the mPFC oscillation. **(H)** Hypothetical Purkinje cell spikes recorded simultaneously with the LFP activity in the mPFC and CA1 shown in **(G)**. The rate modulation of this hypothetical Purkinje cell shows a significant increase in spike firing when the phase difference between mPFC and CA1 oscillations reaches values around 270°. **(I)** Phase histogram of real Purkinje cell simple spike activity. The histogram shows spike activity as a function of mPFC-CA1 phase differences at 11 Hz. The simple spike activity of the Purkinje cell in this example was significantly modulated as a function of mPFC-CA1 phase difference, with a preference of 288.7°. **(J)** Same data as in **(I)** represented in polar coordinates. Vectors composed of the angular value 4 and the magnitude of the spikes per sample were summated to determine the angular preference of Purkinje cell activity. The resultant vector magnitude was taken to quantify the degree of modulation and tested against surrogate results for statistical significance (from McAfee et al., 2019).

Encoding of the oscillatory phase of a cortical region, and calculation of phase difference between two co-active cortical regions, are capabilities that would ideally enable the extraction of the neuronal context associated with a given task. Results from our own studies show that Purkinje cell simple spike activity in cerebellar lobulus simplex (LS) and Crus I of awake mice does indeed represent the instantaneous phases and the phase differences between LFP oscillations in the mPFC and the dorsal hippocampal CA1 region (dCA1) (McAfee et al., 2019). Crus I and LS Purkinje cells differed in their representation of instantaneous phases. In Crus I, Purkinje cells mostly represented the phases of delta oscillations in mPFC and dCA1. In LS, Purkinje cells also represented the phase of delta oscillations, but also the phases of high gamma oscillations in mPFC and dCA1 (Figure 3). Interestingly, phase differences between mPFC and dCA1 oscillations were represented equally in both cerebellar lobules for all major frequency bands of neuronal rhythms (delta, theta, beta, and gamma) (McAfee et al., 2019; Figure 3). The mPFC and dCA1 are known to show modulations of coherence in the context of spatial working memory tasks (Gordon, 2011; Spellman et al., 2015), suggesting a potential involvement of the cerebellum in the modulation of coherence and the associated spatial working memory task.

How does the cerebellar network derive information about oscillatory phase and phase difference at distinct frequencies from the inputs it receives? The largest descending excitatory input to the cerebellar cortex is conveyed *via* neurons in the pontine nuclei that project MFs that synapse with granule cells (GCs) in the cerebellar cortex. Pontine afferents appear to be arranged in such a way as to convey the aggregate activity level of the neuronal field from which they originate. These projection neurons have dense but local dendritic arbors and mutual synaptic connection with neighboring corticopontine neurons (Morishima et al., 2011), and do little to spatially or temporally transform the excitation they receive.

For example, motor cortical efferents remain somatotopically arranged, but non-specific in their synapses – their axons forming numerous branches, with neighboring projections terminating on interlaced fields in the pons (Brodal and Bjaalie, 1992; Schmahmann et al., 2004). This results in a sort of spatial smoothing, and in some cases a mixing of cortical sources, as neuronal signals are transferred from cortical to pontine somatotopy. Pontine inputs from other regions appear to follow the same arrangement.

**FIGURE 4:**
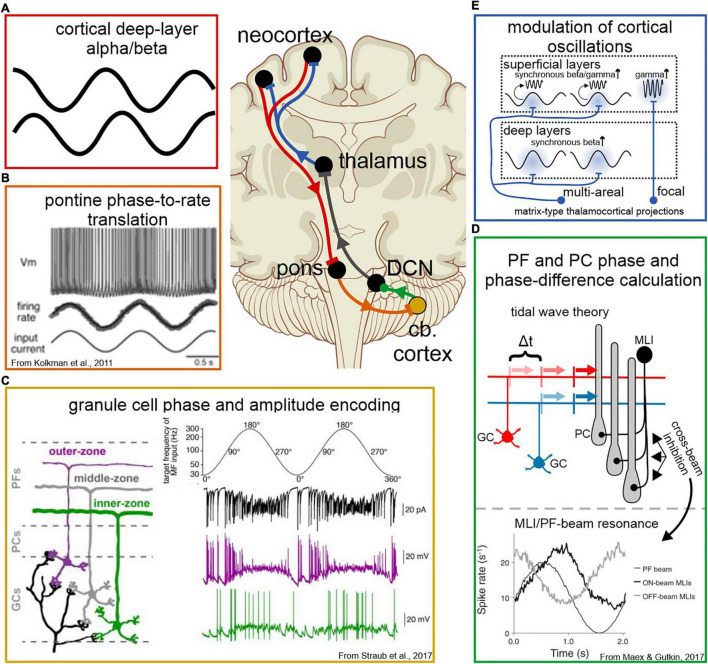
Cellular and network mechanisms of oscillatory encoding and modulation in the cortico-cerebello-cortical circuit. Panel labels are color-coded according to where in the circuit a modulation of the neuronal signal occurs, corresponding to the schematics in the top-center. **(A)** Cortico-cerebellar signals originate in the deep layers of the neocortex, where alpha and beta oscillations predominate. **(B)** Pre-cerebellar neurons in the pons translate a dynamic current input into rate in a linear fashion, thereby translating oscillatory current into a rate code. **(C)** Deep and superficial GCs respond preferentially to different phases of the ponto-cerebellar signal, thereby encoding both phase (*via* time) and amplitude (*via* GC depth) of oscillatory input. **(D)** Phase and phase difference of oscillatory activity is decoded by Purkinje cells, *via* two potential mechanisms. Top: tidal wave theory proposes that a phase difference in a band-limited frequency range can be calculated as a time difference along slow-conducting parallel fibers. Each parallel fiber conveys information about the phase of one cerebral oscillation, and together convey information about the phase relationship of their inputs. Two inputs offset by Δt would arrive simultaneously at the Purkinje cell dendritie. Bottom: simulations show that rhythmic excitation can generate network resonance across parallel fiber beams with a phase shift, due to cross-beam inhibition from MLIs. Rhythmic excitation could augment Purkinje cell responses to input across parallel fiber beams, thereby providing a means to calculate phase differences that are too great to be accounted for in parallel fiber conduction length. **(E)** Feedback to the cortex conveyed *via* thalamocortical projections. Multi-areal matrix-type projections target superficial and deep layers in multiple cortical areas, likely inducing simultaneous beta oscillations that facilitate simultaneous gamma bursts in targeted regions. Focal matrix-type projections preferentially target the superficial layers, suggesting a role in spatially selective augmentation of gamma responses during the bottom-up flow of information.

Despite the diversity of function in the pontine nuclei, pre-cerebellar neurons universally translate their input current into a rate code in a linear fashion (Kolkman et al., 2011; Figure 4B). Consequently, oscillatory population activity from the cortex is received by pre-cerebellar neurons in the pons and immediately transformed into phase information *via* firing rate. [Inversely, sustained DC current drives neuronal spiking activity with irregular intervals, making pontine neurons effective responders to oscillatory input, but ineffective generators of sustained oscillatory output (Schwarz et al., 1997).] Recent studies show that GCs receiving this appear to be biophysically tuned to different phase information within this input as well – along the depth of the GC layer, neurons respond preferentially to inputs of increasing frequency, thereby forming a gradient tuned to distinct phases within the ponto-cerebellar signal (Straub et al., 2020; Figure 4C). Parallel fibers also exhibit a depth-dependence in conduction velocity, with deeper GCs conducting action potentials at a higher velocity (Straub et al., 2020). Modeling showed that these GC properties together led to more precise Purkinje cell responses to a given spike-frequency-modulated MF input (Straub et al., 2020). Altogether, this suggests a role for GCs in isolating phase and amplitude components from the cortico-cerebellar signal before conveying this information to Purkinje cells for temporal comparison.

Interestingly, at least for phase differences of a brief time interval, the cerebellar cortical network architecture is uniquely designed to “calculate” phase difference from oscillatory fiber activity arriving from two different structures (Figure 4D). A phase to phase-difference transform occurs along the slow-conducting parallel fibers in a mechanism first proposed by Braitenberg and Atwood (1958) and Braitenberg et al. (1997) as the “tidal wave hypothesis.” Phase differences between oscillations at any given frequency can be expressed in terms of temporal delays. MFs providing inputs that are phase-locked to oscillations at their respective cerebral cortical site of origin, excite neighboring GCs with delays that are proportional to phase differences between cerebral cortical oscillations. As the spike responses elicited in the GCs propagate along the slow conducting GC axons, the parallel arrangement of these fibers uniquely allows for the asynchronous activity to realign to a synchronous volley of inputs to the two-dimensional dendrites of Purkinje cells (Figure 4D). During periods of robust oscillation, an array of Purkinje cells could passively encode a range of phase relationships expressed by their inputs, allowing the timing of the teaching signal(s) from the climbing fiber pathway to help distinguish contextually meaningful phase relationships for synaptic modification. That the cerebellar network can indeed transform sequential input arriving at the GC layer into synchronous volleys of parallel fiber spikes and elicit sequence specific Purkinje cell responses was shown in a series of *in vitro* experiments by one of us (Heck, 1993, 1995, 1999; see also Braitenberg et al., 1997).

Within this framework, it is important to consider frequency specificity of MF inputs as an important component of the cerebrocerebellar pathway. Cortico-pontine input is driven by neurons in cortical layer V, which primarily carry sub-gamma frequencies (Castro-Alamancos, 2013; Bastos et al., 2018; Figure 4A). For larger phase difference calculation for lower (sub-gamma) frequencies, network resonance properties likely also play a role (Figure 4D).

Interestingly, the cerebellar Golgi cell network, which is connected *via* gap junctions, seems ideally designed to prevent large scale synchronization of the cerebellar input layer in response to rhythmic MF activity (Vervaeke et al., 2010). Gap junctions connecting Golgi cells have unique low pass filtering properties, permitting the propagation of the slow after-hyperpolarization component of an action potential while the fast, depolarizing portion has little to no effect on the membrane potential of neighboring Golgi cells (Vervaeke et al., 2010). This results in a functional lateral inhibition and desynchronization of Golgi cell network activity, allowing rhythmic inputs to remain separated in space and frequency.

Cerebellar network modeling suggests that molecular layer interneurons (MLIs; basket cells and stellate cells) impart circuit resonance that would be consequential for the frequency specificity of encoding phase information (Maex and Gutkin, 2017). In these models, MLI inhibition is shown to set an optimal frequency of resonance that can be varied with the strength of inhibition, potentially providing a mechanism for fine tuning the frequency specificity of the cerebellar response to broad band inputs (Maex and Gutkin, 2017). These same network properties also generate on-beam and off-beam excitation/inhibition cycles that are phase-shifted (Maex and Gutkin, 2017). The full implications of this remain to be explored *in vivo*, but nevertheless provide a potential mechanism for oscillation “memory” as well as prediction within a resonating network, depending on whether the phase is shifted in a positive or negative direction, respectively (Figure 4D). In this scheme, resonance can occur along separate beams in a phase-shifted manner, remaining mutually reinforcing while augmenting Purkinje cell responses across beams in a phase-relationship specific manner, providing a mechanism for phase-relationship encoding at lower frequencies.

Selective drive of deep cerebellar nuclei (DCN) neurons is the final step in the pathway for the generation of feedback to the cortex. There are four main synaptic influences that determine the activity of DCN neurons: inhibitory input from Purkinje cells, excitatory inputs from collaterals from MFs and climbing fibers, and finally synaptic inputs from other neurons within the DCN network (Perciavalle et al., 2013). The inhibitory influence of Purkinje cells dominated early theories implying cerebellar cortical suppression of DCN activity (e.g., Houk, 1991) despite experimental evidence to the contrary, which suggested a more complex reality (McDevitt et al., 1987). To this date surprisingly little is known about how the interacting synaptic inputs determine the activity of DCN projection neurons (Perciavalle et al., 2013). One of the reasons why the DCN networks and neuronal properties are still poorly understood may be the fact that the physiological properties of the DCN neurons do not easily correspond to morphology, and that there is no reliable way to identify cell types based on extracellularly recorded spike shapes or spike activity patterns (Canto et al., 2016). *In vitro* studies suggest that synchronous Purkinje cell activity is an effective mechanism for controlling DCN activity (Gauck and Jaeger, 2000; Person and Raman, 2012) and task specific synchronized Purkinje cell activity has been observed *in vivo* (Heck et al., 2007). There is however, thus far no demonstration of Purkinje cell synchrony modulating DCN firing in a behaving animal. Observation of DCN activity during behavior show a gradual rate modulation on time scales related to the ongoing behavior, suggesting that rate modulated inputs are driving the changes (Thach, 1970; Lu et al., 2013). Additional experiments are needed to determine the role of synchronized inputs in the control of DCN output. An important property of synchronized inhibition is its ability to induce precisely timed spike activity in the DCN (Gauck and Jaeger, 2000; Person and Raman, 2012), which may play a role in the transmission of phase resetting signals from the DCN to thalamus.

### The Cerebellum Transmitting: Cerebellar Coordination of Cerebral Activity

How would cerebellar output influence the coherence of oscillations in two cerebral cortical areas? The thalamus is believed to play a key role in the coordination of cerebral oscillations (Jones, 2001), including the modulation of their coherence (Guillery, 1995; Destexhe et al., 1999; Saalmann, 2014), and notably between the mPFC and dorsal hippocampus (Hallock et al., 2016). Generally, cerebellar outputs terminate on several thalamic nuclei, which contain relay neurons that in turn project throughout the cerebral cortex. Subtypes of thalamic relay neurons can be defined based on which of the cortical layers they target, as these different targets suggest a different influence on cortical activity. A subtype of relay neuron known as matrix-type is thought to play a key role in the modulation of cerebral oscillations (Jones, 2001), and is characterized by widespread lateral axonal arborization in the superficial neocortical layers (Clasca et al., 2012) where gamma oscillations are most prominent. Matrix-type neurons are common in the intralaminar and mediodorsal thalamic nuclei (Clasca et al., 2012), which are thought to have a particularly important role in the coordination of cerebral cortical oscillations, and which receive excitatory input from the cerebellum (Aumann and Horne, 1996a,b; Melik-Musyan and Fanardjyan, 1998; Saalmann, 2014). Matrix-type relay neurons can be further divided into focal- and multi-areal-targeting groups, which (as the name implies) form dense terminals in either one or multiple cortical regions (Clasca et al., 2012; Figure 4E). Interestingly, focal matrix-type relay neurons tend to synapse in the superficial layers exclusively, whereas multi-areal matrix-type neurons target cortical layer V as well (Clasca et al., 2012). Simultaneous excitatory drive to layers I, II/III, and V has been proposed as a mechanism for the generation of beta oscillations in the cortex (Sherman et al., 2016), suggesting that these neurons might modulate inter-areal gamma coherence *via* the induction of gamma-enhancing beta events in multiple regions simultaneously. Many different means of cortical modulation are possible based on cerebello-thalamo-cortical anatomy, yet the exact mechanism(s), or combinations therein, of cerebellar coordination of cerebral cortical oscillations remain to be determined.

It is important to note that the mechanism we propose does not require synchronization of rhythmic neuronal activity between the cerebellum and cerebral cortex. Synchronization of cerebral and cerebellar rhythms have been observed in animals and humans (Ros et al., 2009; Cheron et al., 2016) and have been suggested to reflect ongoing cerebrocerebellar interaction (Cheron et al., 2016). The mechanism we propose here does not require synchronized oscillations between the cerebral and cerebellar cortex. We predict that that cerebellum continually monitors the phase differences between oscillations in two cerebral cortical structures to detect and correct deviations from the optimal phase difference, based on the specific task and the structures involved. Rhythmic Purkinje cell activity synchronized with the cerebral cortex would not necessarily interfere with this function but at the same time the rhythm would not carry any information relevant to the task.

**FIGURE 5:**
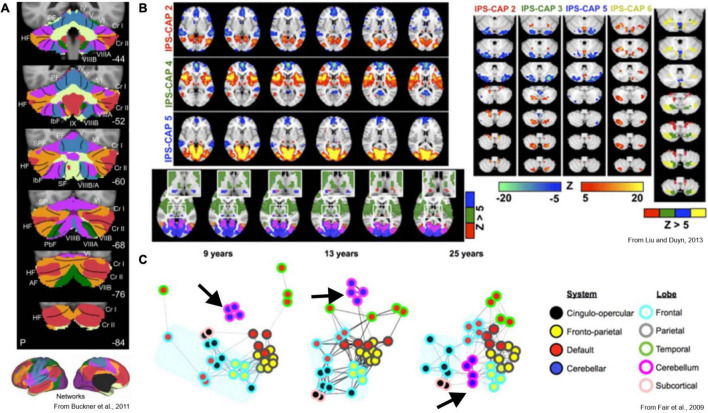
Key functional imaging studies of cerebrocerebellar interaction. **(A)** Voxel-to-network mapping of cerebellar relationship to cerebral intrinsic networks. Most of the cerebellum is most-strongly linked to association and cognitive cerebral areas. **(B)** Co-activation pattern analysis identifies recurring spatial patterns of co-activation in the brain. Left: three unique cerebral co-activation patterns involving the intraparietal sulcus are shown. Lower panel shows unique thalamic foci associated with each pattern as well. Right: corresponding cerebellar activations. Focal activation of cerebellar cortex is linked to complex patterns of co-activation across distributed cerebral cortical networks. The non-overlapping foci suggests a voxel-to-network mapping of cerebellar activity to cortical networks is insufficient to describe the cerebellum’s role in distributed brain networks. **(C)** Maturation of brain networks over the course of development. Black arrow indicates cluster of cerebellar nodes at each developmental stage. Early in development, cortical areas are functionally linked to their nearest anatomical neighbors, and the cerebellum has no functional link to the cortex. Once mature however, the cerebellum acts as a hub between distributed functional networks in cortex.

Further clues as to the cerebellar role in the spatiotemporal organization of cerebral cortical activity can be gleaned from functional imaging studies. Resting state fMRI measurements can be used to identify intrinsic cerebral cortical networks that mimic the regional activation observed during various tasks and at rest. Virtually all functional networks (except visual) (Schmahmann et al., 2019) exhibit robust representation in the cerebellum (Buckner, 2013; Guell et al., 2018a; Marek et al., 2018; Figure 5A), with seemingly similar roles of the cerebellum in task and rest conditions (Schmahmann et al., 2019). The cerebellar representation of resting-state networks contains redundant functional domains in a center-out pattern that resembles the pattern of bifurcated pontocerebellar axonal targeting in rodents (Biswas et al., 2019). The functional relationship between cortex and cerebellum appears this way in resting-state studies that measure steady-state connectivity, but different patterns emerge when the assumption of stationarity is dropped. For example, one study examined which areas of the brain were co-active with the intraparietal sulcus, an association region considered critical for the integration of multisensory information for spatial processing. Interestingly, this region did not co-activate with a single region of the cerebellum, but instead co-activated with several non-overlapping cerebellar regions, each representing which other cortical region(s) were simultaneously active (Liu and Duyn, 2013; Figure 5B). This shows that specific focal activations in the cerebellum correspond to distributed spatial patterns of cerebral cortical co-activation, suggesting that selective inter-areal communication is established between distributed networks in the cerebral cortex when certain cerebellar regions are active. The directionality of this relationship is not known, however, and may represent encoding of cerebral co-activation by the cerebellum, induction of cerebral co-activation by the cerebellum, or an interplay of the two. Investigation of lag between cerebral cortical and cerebellar BOLD signals suggests the former, but the timescale of fMRI is very slow, and the fact that cerebellar BOLD is driven primarily by GC layer input (Diedrichsen et al., 2010) make it difficult to preclude the latter.

The development of whole-brain networks seen in fMRI, especially how the cerebellum is integrated into them, also suggests that the cerebellum could function as a central hub for communication between major cerebral cortical areas (Fair et al., 2009; Figure 5C). Early in neural development, intrinsic cortical networks are poorly defined, with each cortical region only exhibiting correlated activation with its immediate neighboring regions (Fair et al., 2009; Power et al., 2010; Kundu et al., 2018). At this stage, the cerebellum does not appear to share substantial functional links with any cortical regions–in this regard, we are referring here specifically to functional links that would have direct resemblance to mature cognitive function. As the brain reaches circuit maturity, intrinsic spatial patterns emerge in the cerebral cortex, forming distributed networks with correlations that are defined functionally rather than anatomically. At this stage, the cerebellum becomes so embedded into the network structure that it seemingly acts as a hub for the coordination of communication between the distributed cortical networks (Fair et al., 2009; Kundu et al., 2018). Additionally, the regions of the cerebellum with the highest inter-subject variance in functional mapping were those that correspond to cerebral cortical areas related to executive function and cognition (Marek et al., 2018). Altogether, this evidence suggests a number of things: that the cerebrocerebellar relationship maintains coordinated inter-areal communication between functionally defined cortical regions, that focal cerebellar activation corresponds to spatially selective cerebral co-activation, and that these spatial relations that come to define cerebral cortical network organization are learned or acquired over the course of development. We argue that these findings strongly support the idea that the cerebellum integrates information from cerebral cortical activity and teaching signals from the inferior olive to adaptively co-activate regions and establish spatially selective coherence, thus leading to meaningful integration within and across cerebral cortical networks over the course of development. Importantly, this new view we present here not only explains the observed patterns of co-activity in the adult cerebrocerebellar system but provides a framework for the investigation of developmental disorders that are known to involve the cerebellum, such as ASDs and schizophrenia.

Compared to fMRI, electroencephalography (EEG) captures brain activity with much lower spatial but far higher temporal resolution, including frequencies in the gamma range (Freeman et al., 2003). EEG has been applied to investigate cerebellar influence on cerebral cortical activity using non-invasive transcranial magnetic stimulation (TMS) to stimulate the cerebellum (for a recent review see Fernandez et al., 2020). While most cerebellar TMS-EEG studies report on evoked potentials in the cerebral cortex, some also investigated oscillatory activity. Findings from these latter studies showed that cerebral cortical oscillations are modulated by TMS applied to the cerebellum. For example, Farzan et al. (2016) applied intermittent theta burst stimulation (iTBS) to the vermis and the Crus I/II region of the right hemisphere of the posterior cerebellum in healthy adults. Post-stimulation power spectral analysis showed an increase in power of beta to low gamma oscillations in frontal and parietal regions following vermal stimulation, and a global reduction in theta and an increase in high gamma oscillations in fronto-temporal areas following stimulation of the hemisphere (Farzan et al., 2016). The spatial arrangement of these findings is consistent with cerebrocerebellar functional connectivity patterns based on fMRI activity maps (Buckner et al., 2011). Similarly, application of high frequency repetitive transcranial magnetic (rTMS) stimulation of the cerebellum combined with EEG revealed a stimulation-site specific modulation of gamma power in frontal cortical regions (Schutter et al., 2003). Stimulation of the vermis resulted in a shift of gamma power from left frontal to right frontal dominance while stimulation of control sites in the occipital cortex and cerebellar hemisphere did not induce this effect (Schutter et al., 2003). Du et al. (2018) were able to show that cerebellar TMS stimulation increased synchrony between left and right prefrontal areas within the theta to gamma frequency range. What sets their study apart is that they were also able to show that cerebellar-evoked increase in bilateral prefrontal synchrony was associated with better working memory performance, linking cerebellar modulation of cerebral cortical oscillations to cognitive function (Du et al., 2018). These studies thus show that activity in specific cerebellar subregions can influence cerebrocortical neuronal dynamics in multiple frequency bands with regional specificity, and that this influence can be linked to cognitive processes.

### Cerebellar Involvement in Hippocampal–Prefrontal Interactions

Cerebellar involvement in cognitive functions and cognitive disorders that are associated with cerebellar neuropathology involves cerebellar interactions with frontal cerebral cortical areas (Ramnani, 2006; Schmahmann et al., 2019; Wagner and Luo, 2019). More recently, essential spatial functions, such as spatial coding by place cells or spatial memory have been shown to require an intact cerebellum (Tomlinson et al., 2014; Lefort et al., 2015, 2019). Accordingly, trans-neuronal tracing showed projections from cerebellar vermal lobule VI and hemisphere lobule Crus I to the dorsal thalamus (Watson et al., 2019). Connections between the hippocampus and Crus I are notable in the context of cerebellar cognitive function, because Crus I also has reciprocal connections with the prefrontal cortex (Middleton and Strick, 2001), which have recently been directly linked to the control of social behavior in mice (Kelly et al., 2020). The prefrontal cortex and dorsal hippocampus are jointly required for spatial working memory function in rodents (Jones and Wilson, 2005; Benchenane et al., 2011; Wirt and Hyman, 2017; Negron-Oyarzo et al., 2018) and their connection with the cerebellum may help explain findings of cerebellar involvement in spatial orientation (Burguiere et al., 2005; Rochefort et al., 2011) and spatial working memory (Tomlinson et al., 2014).

To determine the physiological nature of hippocampal cerebellar interactions, Watson et al. (2019) implanted mice with recording electrodes in the dorsal hippocampus, vermal lobule VI and Crus I. They then trained the mice in a simple goal-directed behavior, requiring the mice to traverse a linear path to receive a reward consisting of an electrical stimulation of the medial forebrain bundle (Carlezon and Chartoff, 2007) at the end of the path (Watson et al., 2019). As mice improved their performance of this goal-directed behavior, coherence of theta oscillations (6–12 Hz) between the dorsal hippocampus and Crus I selectively increased (Watson et al., 2019), suggesting that the communication between Crus I and dorsal hippocampus involves task related coherence of neuronal oscillations (Watson et al., 2019).

## Implications for Cognitive Disorders

Cerebellar coordination of neuronal communication predicts that cerebellar pathophysiology would result in deficits in neuronal communication between brain areas and that those deficits would be detectable in measurements of functional connectivity. This should be testable in brain disorders that have a high likelihood of cerebellar neuropathology, such as ASDs and schizophrenia. Interestingly, a hypothesis of brain-wide dysconnection (disordered functional connectivity between brain structures) as a major underlying cause was advanced for both ASDs (Just et al., 2004, 2012; Wass, 2011) and schizophrenia (Stephan et al., 2009; Pettersson-Yeo et al., 2011; Tu et al., 2012). There is, however, no agreement as to the causes of the dysconnectivity; however, they could conceivably occur at the anatomical or functional levels, since such circuit-based disorders often arise due to a combination of circuit miswiring, neuronal degeneration, and functional abnormalities.

Additionally, the inevitable surgical damage to the cerebellum, that occurs during medulloblastoma resection in the posterior fossa region, is known to cause broad cognitive, emotional, and behavioral deficits, particularly in the case of disruption of the cerebellar output tract in children (Morris et al., 2009). The underlying neurobiological causes of this disorder (known as Cerebellar Mutism Syndrome or Posterior Fossa Syndrome) remain unclear, but this disorder highlights the importance of cerebellar output in the development and maintenance of cerebral activity to support normal cognitive function.

### Coherence/Functional Connectivity Abnormalities in Autism Spectrum Disorders

Frith (1997) suggested that many of the perceptual and attentional abnormalities in ASDs could be interpreted as “weak central coherence,” which she defines as a reduction in the contextual integration of information and a bias toward local rather than global processing, i.e., the inability to integrate pieces of information into a coherent whole. Other authors attributed weak central coherence to an impairment of “temporal binding” between local networks, whereas temporal binding within local networks was presumed to be intact or possibly even enhanced (Brock et al., 2002). Animal studies offer some clues as to the neuronal mechanisms underlying this type of deficit, and how it may result from cerebellar dysfunction. As discussed previously, this type of impairment is analogous to what is observed in the sensorimotor system of rats when cerebellar output nuclei are inhibited, with the coherence between sensory and motor cortices disrupted while local processing remains intact (Popa et al., 2013). Another recent study showed how ASD-like behavior in mice is linked to activity in specific cerebello-thalamo-prefrontal cortical projections (Kelly et al., 2020). Viral tracers were used to drive expression of channelrhodopsin or archaerhodopsin in the polysynaptic projections to mPFC originating from the right Crus I. Increased activity in these terminals *via* optical stimulation increased ASD-like behaviors, while optical inhibition decreased them. Increased activity in this pathway is thought to be linked to the loss of Purkinje cells in the cerebellar cortex that occurs in ASD (Fatemi et al., 2012), resulting in persistent excitatory output. With regard to CTC, dysfunction or loss of Purkinje cells likely results in less opportunity for selective spatiotemporal synchronization, since excitatory output from the cerebellum is normally modulated in response to task-relevant patterns of cerebral activity. Selective synchronization occurs when activation in selected neocortical regions stands out from a background level of neuronal activity, which becomes increasingly difficult as the background level of activity is increased.

In a study of resting state EEG activity that focused specifically on coherence in the low frequency (0.5–3.5 Hz) range, Barttfeld et al. (2011) reported reduced long-range and increased short-range coherence in individuals with ASD. The same study showed that the magnitude of the coherence deficit compared to control subjects scaled with the severity of the ASD phenotype (Barttfeld et al., 2011). Murias et al. (2007) also used EEG recordings and reported increased local and reduced long-distance coherence in individuals with ASD compared to typically behaving control subjects. Task related functional connectivity, measured with fMRI, was found to be reduced in the visual system of patients with ASD during a task testing working memory of faces (Koshino et al., 2008).

Dinstein et al. (2011) investigated interhemispheric synchronization in toddlers with ASD while they were sleeping in an fMRI. They reported significantly reduced interhemispheric synchronization between language areas and showed that the magnitude of the synchronization was negatively correlated with ASD severity (Dinstein et al., 2011). Supekar et al. (2013) also used fMRI to study an older group of children while they were awake and found that the brains of children with ASD showed brain-wide hyperconnectivity, with the degree of hyperconnectivity predicting the severity of social behavior deficits. Another study by Oldehinkel et al. (2019) examined cerebrocerebellar fMRI connectivity more directly and found that the subjects with ASD exhibited an increase in connectivity between the cerebellum and primary sensory and motor networks. At the same time, the functional connectivity within these networks was abnormally low, with the degree of the connectivity deficit correlated with the severity of symptoms such as sensory processing, repetitive behaviors, and social impairments.

While it is becoming increasingly clear that the cerebellum plays an important role in the development of cerebral functional networks, studies exploring the development of cerebrocerebellar functional connectivity in ASD are lacking. In the meantime, studies of cerebellar cortical development offer some clues as to a functional role of the cerebellum in ASD etiology. Focal gray matter volumes have been found to correlate with performance in specific cognitive domains (Moore et al., 2017) for typically developing children, as well as symptom severity in ASD (D’Mello et al., 2015). Most dramatically, D’Mello et al. (2015) showed that underdevelopment of the right Crus I and Crus II was common in subjects with ASD and associated with higher severity of all symptoms assessed by the Autism Diagnostic Observation Schedule. The authors noted that Crus I/II is functionally connected with the prefrontal and parietal cortices, which are shown to have decreased inter-areal connectivity (hypoconnectivity) in ASD (Washington et al., 2014). This suggests that abnormal development of the gray matter in Crus I/II causes a deficit of selective synchronization between its target nodes, and that this loss of selective synchronization may be a key driver of cognitive and behavioral deficits affecting individuals with ASD.

While the results of these studies show some variability, they consistently show that the brains of individuals with ASD have deficits in intrinsic functional connectivity. Interestingly, these results show an apparent tendency toward low-complexity functional network organization in subjects with ASD (Lai et al., 2010; Rudie et al., 2013) – reflecting either excessive segregation or excessive integration of function (Lord et al., 2017). Such deficits are consistent with Frith’s theory of ASD and would be predicted to result from cerebellar pathology and/or pathophysiology if the cerebellum is tasked with the coordination of selective communication between brain areas.

### Coherence/Functional Connectivity Abnormalities in Schizophrenia

Schizophrenia or schizophrenia-like symptoms have long been associated with cerebellar neuropathology (Weinberger et al., 1980; Jurjus et al., 1994; Martin and Albers, 1995). A recent study with a sizable and diverse cohort of 983 schizophrenia patients and 1349 healthy controls used MRI to evaluate structural changes in the cerebellum and cerebral cortex (Moberget et al., 2018). In agreement with earlier studies, Moberget et al. (2018) reported a significant reduction of cerebellar gray matter volume in schizophrenia patients compared to control subjects. The largest volume reduction in the cerebellum patients was found in LS, Crus I and Crus II (Moberget et al., 2018). Those same cerebellar areas have previously been shown to be functionally connected with frontoparietal cerebral cortical areas (Buckner et al., 2011). Moberget et al. (2018) found a significant correlation between cerebellar gray matter volume and frontoparietal cortical thickness. Interestingly, this correlation that was strongest in schizophrenia patients, suggesting that the underlying disease jointly affects the cerebellum and cerebral cortex (Moberget et al., 2018).

Karl Friston and Uta Frith proposed dysconnection as a cause of schizophrenia (Friston and Frith, 1995; McGuire and Frith, 1996; Friston, 1999). Results from imaging studies that evaluate functional connectivity in brains of schizophrenia patients and healthy controls largely support the dysconnection hypothesis. For example, the analysis of resting state functional connectivity MRI (rs-fcMRI) showed that patients had deficits in the default-mode network, the fronto-parietal and saliency networks (Orliac et al., 2013; Chang et al., 2014; Sheffield et al., 2015; Goswami et al., 2020), and had abnormal cerebrocerebellar connectivity (Repovs et al., 2011; Tu et al., 2012; Sheffield and Barch, 2016; Kim et al., 2020). At least one study reported that (Moberget et al., 2018) the severity of schizophrenia symptomatology scaled with the magnitude of the deficits in resting state network connectivity (Orliac et al., 2013). There is currently no agreement on the causes of dysconnectivity. Suggestions include reduced white matter connections but also the possibility of abnormal synaptic plasticity (Stephan et al., 2009; Pettersson-Yeo et al., 2011). The role of the cerebellum we propose here adds a crucial third possibility, suggesting that the deficits in network connectivity in schizophrenia are a consequence of loss of cerebellar coordination of CTC.

In an fMRI study that focused on network interactions, Andreasen et al. (1996, 1998) described a dysfunctional prefrontal-thalamic-cerebellar circuitry in schizophrenia patients and proposed that as a result, schizophrenia patients suffer from “cognitive dysmetria.” The choice of the term “dysmetria” implicates the cerebellum, as that term commonly describes the inability of cerebellar patients to appropriately control the distance of limb or eye movements. There is no clear specification of how dysmetria applies to cognitive processes, but the proposed coordination of communication by the cerebellum, as we propose here, relies on principles of precise temporal coordination by the cerebellum that are otherwise ascribed to cerebellar coordination of movements (Diener et al., 1992, 1993). A cerebellar role in coordinating communication between brain areas, and its failure in the brains of schizophrenia patients offers a possible explanation for the findings of dysconnectivity within the cerebral cortex and between the cerebral cortex and the cerebellum. Failed temporal coordination in motor control results in movement dysmetria because the timing of agonist and antagonist activation and inhibition times are no longer appropriately aligned. Applied to cerebral cortical oscillations, failed temporal coordination results in dysmetria of communication because the timing of phase relationships between communication structures is no longer supporting efficient communication.

There is currently no experimental work that would directly show a deficit in cerebellar coordination of CTC in schizophrenia. However, studies using cerebellar stimulation in schizophrenia patients provide evidence that delta and theta oscillation power, which is reduced in the frontal cortex of patients (Parker et al., 2017), can be restored by rhythmic stimulation of the cerebellum (Singh et al., 2019). The influence of the cerebellum on frontal cortical delta activity was reproduced in rats, where delta-activity in the frontal cortex was reduced after locally blocking D1 dopamine receptors, a model that mimics D1 dysfunction in schizophrenia (Parker et al., 2017). The subsequent delta-frequency optogenetic stimulation of thalamic synaptic terminals of afferents from the lateral (dentate) cerebellar nucleus was sufficient to restore delta activity in the frontal cortex (Parker et al., 2017). In this same study, the rats were trained to perform an interval timing task, estimating interval duration of 3 and 12, and blocking frontal cortical D1 receptors reduced the rat’s performance in the task. Task performance was again rescued by stimulation of thalamic synaptic terminals of afferents from the lateral (dentate) cerebellar nucleus (Parker et al., 2017). Schizophrenia patients receiving theta burst trans-cranial magnetic stimulation reported significant mood elevations and showed improved memory performance (Demirtas-Tatlidede et al., 2010). Using a similar stimulus for the cerebellum and comparing theta and delta frequency stimuli, Singh et al. (2019) showed an increase in theta oscillation power in the frontal cortex of schizophrenia patients, suggesting the modulation of frontal delta/theta range oscillations by the cerebellum as a possible underlying mechanism for the cognitive and affective improvements observed by Demirtas-Tatlidede et al. (2010).

How the cerebellum modulates delta/theta power in the frontal cortex and how cerebellar neuropathology and its related functional pathophysiological defects would result in diminished delta/theta activity in schizophrenia is unclear. However, several studies have shown that the cerebellum modulates dopamine release in the frontal cortex (Mittleman et al., 2008; Rogers et al., 2011). These findings suggest a direct link between deficits in cerebellar function and deficits in frontal cortical dopamine regulation, which is widely regarded to be a key underlying cause of schizophrenia.

## Existing Views of Cerebrocerebellar Interactions

Cerebrocerebellar interactions have mostly been investigated in the motor domain. We agree with the premise brought forth in recent work (Wagner and Luo, 2019; Li and Mrsic-Flogel, 2020) that the cerebrocerebellar interactions in the cognitive domain are likely analogous to how the cerebellum interacts with motor areas. Thus, it is reasonable to ask how views developed for cerebrocerebellar interaction in motor control can be applied to cerebellar cognitive function and specifically, how they relate to the view we propose here. Several recent studies investigating cerebrocerebellar interactions in the context of preparatory activity provide strong evidence for a cerebellar involvement in the generation of preparatory activity in motor cortical areas (Gao et al., 2018; Chabrol et al., 2019; Li and Mrsic-Flogel, 2020). There is general agreement that the cerebrocerebellar connection loop forms the neuronal basis for cerebellar involvement in the generation of preparatory motor activity. Experimental evidence shows that lesioning of either the neocortex or cerebellum disrupts preparatory activity in the other region, indicating that preparatory activity in the two structures is interdependent (Gao et al., 2018; Chabrol et al., 2019; Li and Mrsic-Flogel, 2020). However, the nature of the neuronal interaction exchanged *via* the cerebrocerebellar loop remains unclear. Li and Mrsic-Flogel (2020) suggest that the cerebellum, through supervised learning, recognizes specific patterns of cerebral cortical inputs and in response returns predictive signals to trigger a state transition in the cerebral cortex shaped to minimize errors in the execution of the next movement segment. Supervised learning is a process by which a system maps input patterns to output patterns based on the observation of consistent input-output pairs, with climbing fiber inputs widely believed to provide error signals (Raymond and Medina, 2018). With regard to movement, the cerebellum is thought to encode neuronal signals related to movement commands as well as their sensory consequences in order to learn their relationship and provide feedback to minimize the difference between expectation and outcome. For a recent review see Raymond and Medina (2018). Alternatively, climbing fibers may generate teaching signals that defy the supervised learning paradigm; it has been shown that under certain conditions teaching signals are scalar, and vary with the predictability of a given stimulus. These features of teaching signals are more consistent with a temporal-difference model of reinforcement learning (Ohmae and Medina, 2015; Lawrenson et al., 2016; Hull, 2020). These hypotheses establish a clear purpose for cerebellar feedback *via* the cerebrocerebellar loop but leave unaddressed the spatiotemporal nature of the signals exchanged, and how they might conform with our current understanding of cortical states. In other words, if corticocerebellar signals need to faithfully represent cortical activity states, and cerebellocortical signals need to be designed to reliably guide cortical activity to the next desired state, then the following key questions arise. How is the oscillatory neuronal activity that defines cortical states represented in MF inputs and how can this activity be altered *via* cerebellothalamocortical projections?

A recent study by Wagner et al. (2019) provided important new insights into cerebellar representation of cerebrocortical activity states. For their study, head-fixed mice learned to shift a lever to the left or right for a water reward while the activity of layer V (L5) neurons in the forelimb premotor area and GC activity in cerebellar lobule VI were monitored with 2P-calcium imaging throughout the learning process. As task performance improved, the activity patterns of L5 premotor cortical neurons and that of lobule VI GCs become increasingly similar (Wagner et al., 2019). Cerebrocerebellar interaction during a learned motor task thus ultimately may result in cerebral cortical activity states to be represented in the input layer of the cerebellar cortex. Importantly, this is consistent with other studies showing an increase in functional connectivity between the cerebellum and cerebral cortex during motor learning (Mehrkanoon et al., 2016), suggesting that learning facilitates information transmission between cerebral and cerebellar areas involved in the learned task. Both of the above studies focused on cerebellar interaction with a single cerebral cortical area and in the context of motor control (Mehrkanoon et al., 2016; Wagner et al., 2019). If this mechanism holds true for cerebellar interactions with other cerebral cortical areas, it provides a mechanism for the cerebellum to access activity states in cerebral cortical areas with which it interacts in the context of learning. In a more general sense, we argue that the cerebellum encodes a cortical state based on a signature arrangement of distributed neocortical oscillations, and subsequently generates outputs that drive thalamic neurons to modulate oscillatory activity to achieve the desired new cortical state. Specifically, we propose that cerebellar projections to the thalamus are likely to influence thalamic matrix neurons, which terminate preferentially on inhibitory interneurons in cortical layer I (Cruikshank et al., 2012), which play a key role in the generation and modulation of cortical oscillations, especially gamma rhythms (Atallah and Scanziani, 2009; Cardin et al., 2009).

## Testing the Validity of the Proposed New Role of the Cerebellum

Future animal and clinical (imaging) experiments should be designed to allow the analysis of cerebellar activity and its relationship to coherence between cerebral cortical areas. Currently, all experiments and analyses focus on modulation of activity in individual cerebral and cerebellar areas. The key is to rethink this approach and consider the functional connectivity *via* coherence between cerebral cortical areas as a dependent variable to correlate with cerebellar cortical activity. Human imaging studies lend themselves to this type of analysis but with the limitations that EEG and MEG, which capture fast dynamics, cannot readily access deep cerebellar structures. Magnetic resonance imaging can access activity in brain structures at any location but will only capture slow changes in activation. Animal studies that combine single-unit recordings in the cerebellum, thalamus and cerebral cortex with cell type specific manipulations of cerebrocerebellar connection pathways will be necessary to provide details about the circuits involved, the behaviors affected and the possible influence of neuromodulatory transmitters. The now well documented influence of cerebellar activity on dopamine release in the prefrontal cortex (Mittleman et al., 2008; Rogers et al., 2011) has been suggested to serve reward related functions (Wagner et al., 2017; Carta et al., 2019) but is also likely to influence the power of frontal cortical oscillations in the delta and theta frequency range. Here, we focused our arguments on cognitive function as the most intriguing new role of the cerebellum. However, cerebellar involvement in sensorimotor control is likely to invoke the same principles of task dependent coordination of CTC. After all, cerebellar coordination of coherence in the cerebral cortex was first demonstrated between the primary sensory and motor cortices in rats (Popa et al., 2013) and more recently in the whisker barrel system in mice (Lindeman et al., 2021).

The principle of cerebellar coordination of precisely timed events, as it occurs in the control of muscle contractions to optimize motor coordination, is here applied to the coordination of neuronal oscillations to optimize cerebral cortical communication during cognitive processes. The elegance of this new perspective of cerebrocerebellar interaction lies in its intuitive simplicity that does not require additional assumptions about cerebellar function and can provide a functional interpretation of cerebellar cortical network architecture.

## Data Availability Statement

The original contributions presented in the study are included in the article/supplementary material, further inquiries can be directed to the corresponding author.

## Author Contributions

All authors contributed to the development of the concept. SM and DH wrote and edited the manuscript. YL and RS contributed to writing and editing.

## Conflict of Interest

The authors declare that the research was conducted in the absence of any commercial or financial relationships that could be construed as a potential conflict of interest.

## Publisher’s Note

All claims expressed in this article are solely those of the authors and do not necessarily represent those of their affiliated organizations, or those of the publisher, the editors and the reviewers. Any product that may be evaluated in this article, or claim that may be made by its manufacturer, is not guaranteed or endorsed by the publisher.
